# Transient Anterior Subcapsular Vacuolar Lens Opacities After Trabeculectomy Using a Dispersive Ophthalmic Viscosurgical Device: Report of Five Cases

**DOI:** 10.7759/cureus.76350

**Published:** 2024-12-24

**Authors:** Hiroshi Shimizu, Masaki Tanito

**Affiliations:** 1 Ophthalmology, Shimizu Eye Clinic, Matsue, JPN; 2 Ophthalmology, Shimane University Faculty of Medicine, Izumo, JPN

**Keywords:** anterior subcapsular cataract (asc), dispersive ophthalmic viscosurgical device (ovd), surgical complication, trabeculectomy, transient cataract

## Abstract

Five cases of transient anterior subcapsular vacuolar lens opacities following trabeculectomy using a dispersive ophthalmic viscosurgical device (OVD) performed by the same surgeon were reviewed at Shimane University Hospital from February to June 2024. All cases presented with anterior vacuolar subcapsular lens opacities observed seven days after surgery, which gradually resolved without specific interventions. Detailed surgical records and clinical outcomes were collected, and the findings suggest that these opacities, characterized by their transient and vacuolar appearance, should be recognized as an early complication of trabeculectomy, potentially linked to the use of dispersive OVDs.

## Introduction

Trabeculectomy is a time-tested surgical approach for managing glaucoma, aiming to create a controlled outflow pathway between the anterior chamber (AC) and the subconjunctival space, thus facilitating aqueous humor filtration and reducing intraocular pressure [1,2]. Despite its efficacy, this procedure is not without complications. Common postoperative issues include subconjunctival fibrosis, which can compromise surgical success, and anterior chamber collapse due to excessive filtration, potentially leading to hypotony or shallow anterior chamber [3-5].

To mitigate these risks, the intraoperative use of ophthalmic viscoelastic devices (OVDs) in the AC has been increasingly adopted. OVDs provide structural support to the anterior chamber, stabilize tissues, and may prevent abrupt fluid outflow during surgery, thereby reducing the likelihood of postoperative complications such as hypotony and overfiltration [6,7].

However, the use of dispersive OVDs during intraocular surgery has been implicated as a potential cause of transient, early postoperative vacuolar cataracts [8,9]. This report highlights a specific postoperative phenomenon observed in five cases following trabeculectomy with dispersive OVDs. These patients developed transient subcapsular vacuolar cataracts characterized by distinct morphological changes in the lens.

## Case presentation

This case series includes five instances of transient anterior subcapsular vacuolar lens opacities following trabeculectomy, all performed by the same surgeon (M.T.) at Shimane University Hospital between February and June 2024. A thorough review of surgical records detailing clinical observations and outcomes was undertaken.

The surgical procedure was standardized across all cases. Preoperative preparation involved the administration of pilocarpine hydrochloride drops (Sanpilo 2%, Santen Pharmaceutical, Osaka, Japan) to induce pupillary constriction. The ocular surface was disinfected with povidone-iodine (Isodine solution 10%, Mundipharma K.K., Tokyo, Japan), and sterile surgical eye drapes were applied. Conjunctival cleansing was performed using iodine polyvinyl alcohol (PA-Iodo Ophthalmic and Eye Washing Solution, Nitten Pharmaceutical Co. Ltd., Nagoya, Japan). Topical anesthesia was induced via sub-Tenon injection of lidocaine (Xylocaine 2%, Sandoz Pharma K.K., Tokyo, Japan). A fornix-based conjunctival flap was fashioned in the superior quadrant. Dissection of the conjunctiva and Tenon’s capsule was carried out carefully, and the sub-Tenon space was expanded with blunt-tipped scissors to facilitate bleb formation. A half-thickness scleral flap (3 mm × 3 mm) was created at the limbus. A sponge soaked in 0.2% mitomycin C was placed in the sub-Tenon space for three minutes, followed by thorough irrigation with a balanced salt solution (BSS) to remove residual mitomycin C. A sclerotomy was performed beneath the scleral flap using a punch incision, followed by trabeculectomy and peripheral iridectomy at the same site. The scleral flap was secured with a single 10-0 nylon suture.

To stabilize the AC, a blunt needle was inserted beneath the sutured scleral flap and into the AC, and a viscoelastic agent containing purified sodium hyaluronate and chondroitin sulfate sodium (Shellgan 0.5, Santen Pharmaceutical) was injected until approximately half of the AC volume was filled. The conjunctival flap was closed using a 10-0 vicryl suture, and the integrity of the closure was confirmed intraoperatively.

At the conclusion of surgery, 2 mg of betamethasone sodium phosphate (Rinderon, Shionogi Pharma Co. Ltd., Osaka, Japan) was injected subconjunctivally, and 0.3% ofloxacin ointment (Tarivid, Santen Pharmaceutical) was applied. Postoperative management included topical administration of 1.5% levofloxacin (Levofloxacin, Viatris, Tokyo, Japan) four times daily for three weeks and 0.1% betamethasone (Sanbetason, Santen Pharmaceutical) four times daily for six weeks.

Case 1

**Figure 1:**
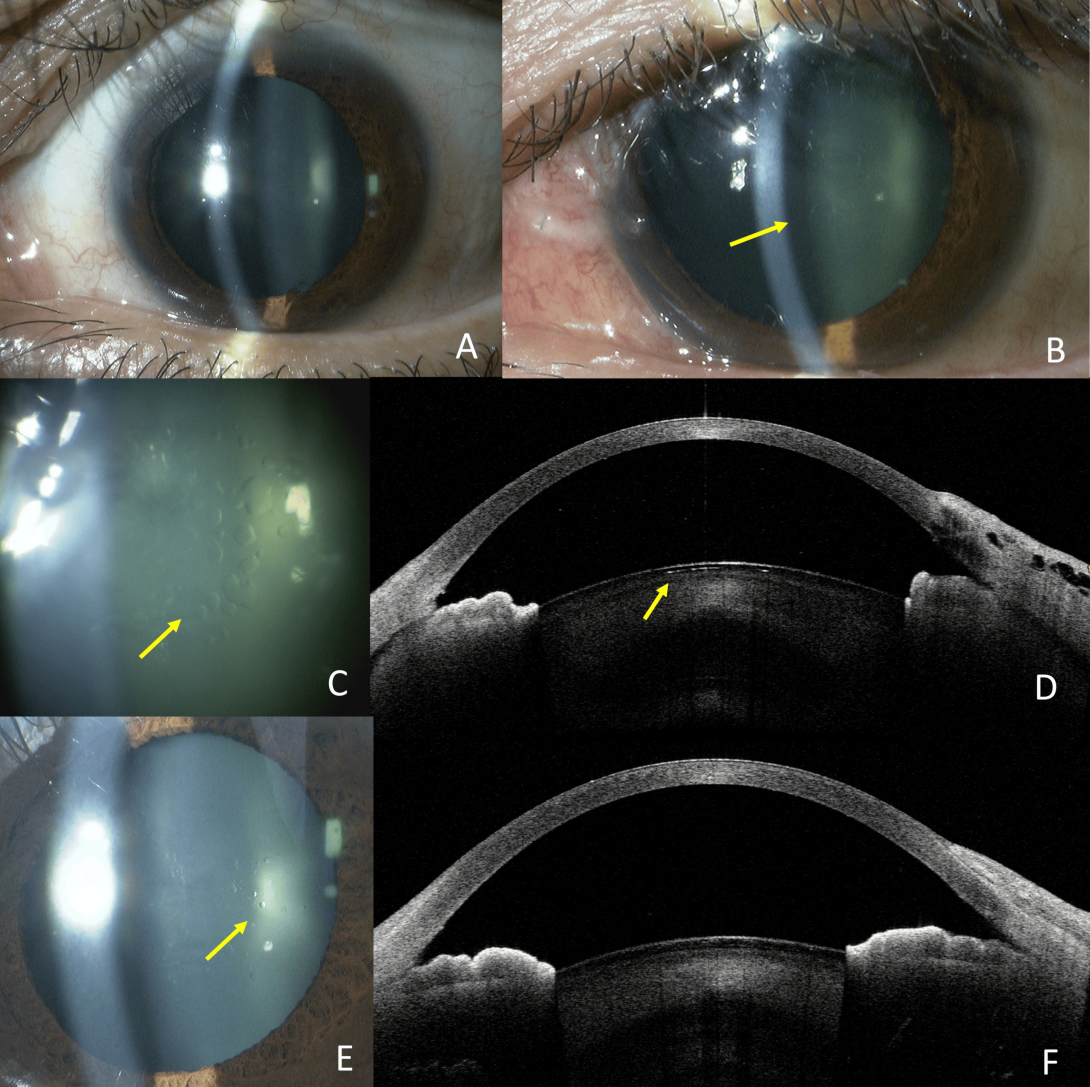
Case 1. Slit-lamp photography and anterior segment OCT imaging. Absence of anterior subcapsular cataract preoperatively (A). POD 7 showed vacuolar anterior subcapsular opacities of the lens (arrow) (B). Opacities on a magnified image of the lesion (arrow) (C). Opacities are detected beneath the anterior lens capsule (arrow) on AS-OCT (D), appearing as small cystic structures characterized by hypointense inner signals and hyperintense outer signals (arrow). At six months postoperatively, the opacity had largely resolved, although some remnants were still visible (arrow) (E). The cystic structures on AS-OCT had completely resolved (F). POD: Post-operative day; AS-OCT: Anterior segment optical coherence tomography

A 42-year-old man underwent trabeculectomy for juvenile open-angle glaucoma in the left eye, which had no preoperative anterior subcapsular cataract (Fig 1A). The best-corrected visual acuity (BCVA) in decimal notation was 0.9, and the patient had a high myopia of -10.0 diopters. The preoperative intraocular pressure (IOP) measured with the Goldmann applanation tonometer was 45 mmHg. On postoperative days (POD) 1 to 3, IOP was 2-3 mmHg, but the AC remained deep. By POD 7, the decimal BCVA had decreased to 0.2, and the IOP was 3 mmHg. Subcapsular vacuolar lens opacity was observed (Fig 1B, 1C). Opacities are detected beneath the anterior lens capsule on anterior segment optical coherence tomography (AS-OCT, Casia 2, Tomey Corporation, Nagoya, Japan), appearing as small cystic structures characterized by hypointense inner signals and hyperintense outer signals (Fig 1D). The IOP stabilized in the mid-teens, and the decimal BCVA partially recovered to 0.4 at six months postoperatively (final visit). Subcapsular vacuoles gradually decreased in size each month and nearly resolved (Fig 1E). Correspondingly, the cystic formations were no longer visible on AS-OCT imaging (Fig 1F).

Case 2

**Figure 2:**
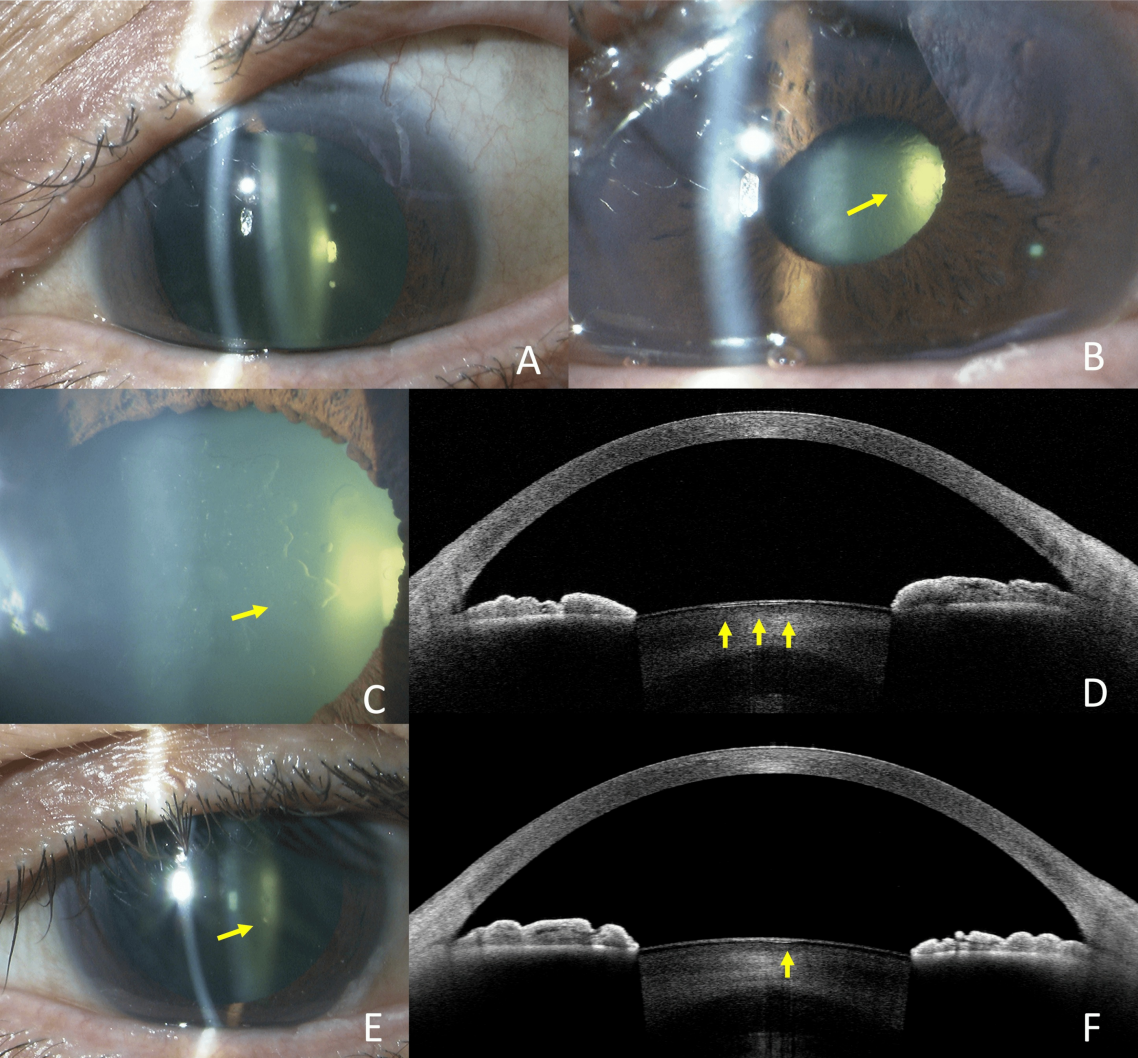
Case 2. Slit-lamp photography and anterior segment OCT imaging. Absence of anterior subcapsular cataract preoperatively (A). POD 7 showed vacuolar anterior subcapsular opacities of the lens (arrows) (B). Opacities on a magnified image of the lesion(arrow)(C). Opacities are detected beneath the anterior lens capsule on AS-OCT (D), appearing as hypointense vacuoles surrounded by a hyperintense capsule as in Case 1 and partially fused(arrows). At four months postoperatively, the opacity had largely resolved, although some remnants were still visible (arrow) (E). The cystic structures on AS-OCT had decreased (arrow) (F). POD: Post-operative day; AS-OCT: Anterior segment optical coherence tomography

A 64-year-old man underwent trabeculectomy for secondary open-angle glaucoma (SOAG) caused by uveitis in the left eye. Preoperatively, there were no anterior subcapsular cataracts (Fig 2A), the decimal BCVA was 0.9, and the patient had myopia of -3.75 diopters. On POD 7, the decimal BCVA had decreased to 0.4, and the IOP was 25 mmHg. Vacuolar lens opacities with partially confluent morphology were observed (Fig 2B, 2C). AS-OCT showed hypointense vacuoles surrounded by a hyperintense capsule as in Case 1 and partially fused (Fig 2D). By four months postoperatively (final visit), the vacuolar lens opacities had significantly diminished, and the decimal BCVA improved to 1.2 (Fig 2E). The cystic structures on AS-OCT had decreased (Fig 2F).

Case 3

**Figure 3:**
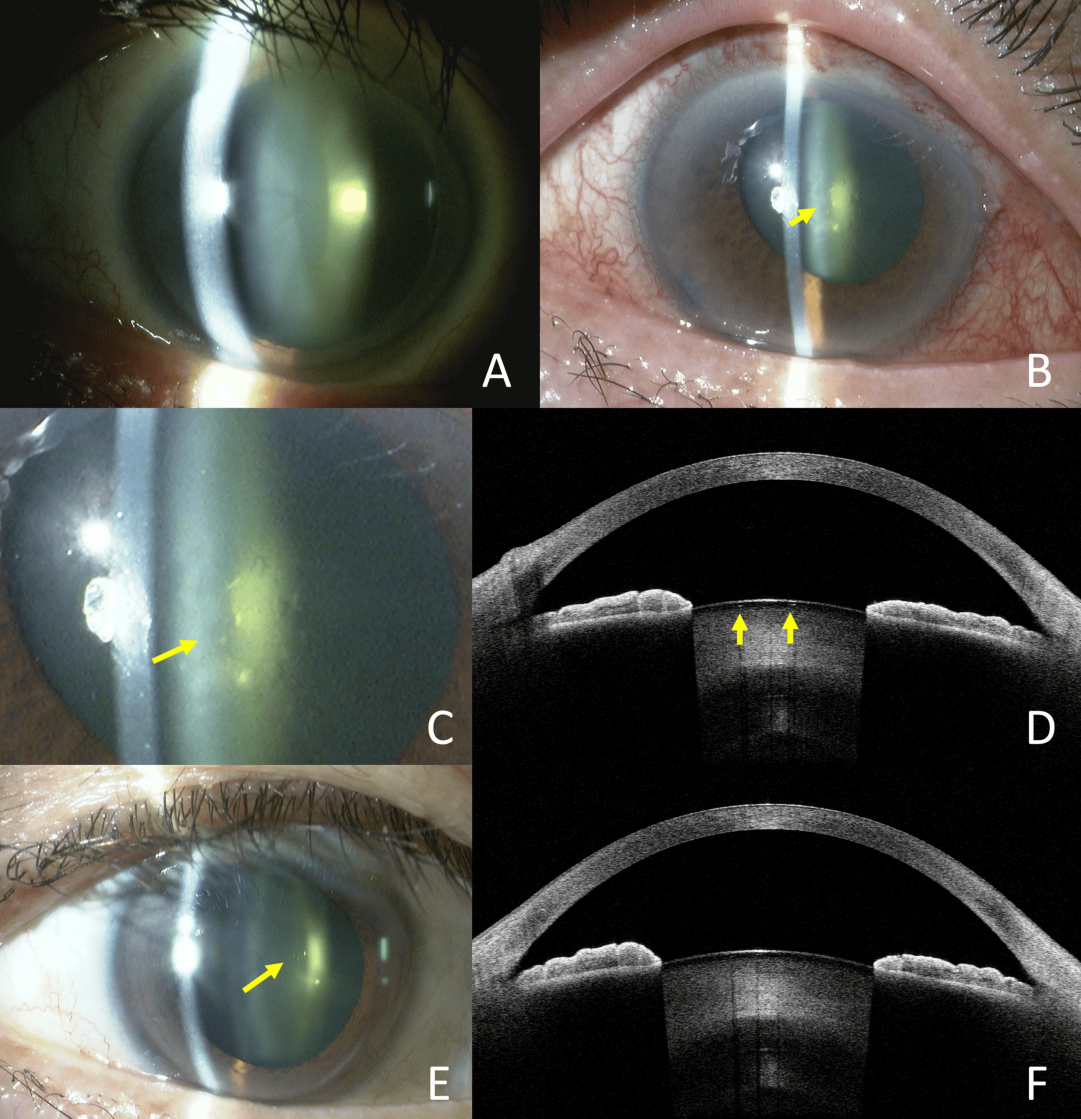
Case 3. Slit-lamp photography and anterior segment OCT imaging. Absence of anterior subcapsular cataract preoperatively (A). POD 7 showed vacuolar anterior subcapsular opacities of the lens (Arrow) (B). Opacities on a magnified image of the lesion (Arrow) (C). AS-OCT showed vacuoles as in Case 1 (Arrows) (D). Eight months after surgery, the opacity had largely resolved, although some remnants were still visible (Arrow) (E). The cystic structures on AS-OCT had completely resolved (F). POD: Post-operative day; AS-OCT: Anterior segment optical coherence tomography

A 65-year-old man underwent trabeculectomy for primary open-angle glaucoma (POAG) in the right eye, which had no preoperative anterior subcapsular cataract (Fig 3A). Preoperatively, the decimal BCVA was 1.2, and the patient had high myopia of -9.25 diopters. On POD 7, the decimal BCVA had decreased to 0.6, and the IOP was 10 mmHg. Vacuolar lens opacities were observed at this time (Fig 3B, 3C). AS-OCT showed vacuoles as in Case 1 (Fig 3D). By eight months postoperatively (final visit), the lens opacities had nearly disappeared (Fig 3E), and the decimal BCVA had improved to 1.2. The cystic structures on AS-OCT had completely resolved (Fig 3F).

Case 4

**Figure 4:**
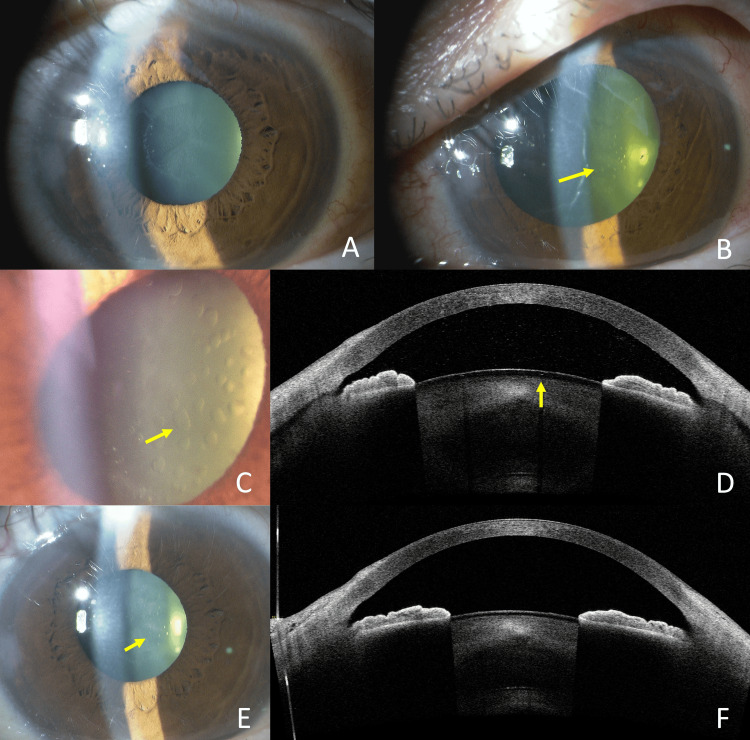
Case 4. Slit-lamp photography and anterior segment OCT imaging. Absence of anterior subcapsular cataract preoperatively (A). POD 7 showed vacuolar anterior subcapsular opacities of the lens (Arrow)(B). Opacities on a magnified image of the lesion (Arrow) (C). Only a few vesicles were detected on AS-OCT (Arrow)(D). The opacity had largely resolved four months after surgery, although some remnants were still visible (Arrow) (E). Vesicles had not been detected on AS-OCT (F). POD: Post-operative day; AS-OCT: Anterior segment optical coherence tomography

A 60-year-old man underwent trabeculectomy for SOAG caused by exfoliation syndrome in the left eye. Preoperatively, there were no anterior subcapsular cataracts (Fig 4A), and the decimal BCVA was 1.2. The patient had a myopia of -4.75 diopters. On POD 7, the decimal BCVA had decreased to 0.2, and the IOP was 3mmHg. Vacuolar lens opacities were observed (Fig 4B, 4C). Only a few vesicles were detected on AS-OCT (Fig 4D). By four months postoperatively (final visit), the vacuolar lens opacities had slightly decreased but were still present (Fig 4E) and had disappeared on AS-OCT (Fig 4F). The decimal BCVA had recovered to 1.2.

Case 5

**Figure 5:**
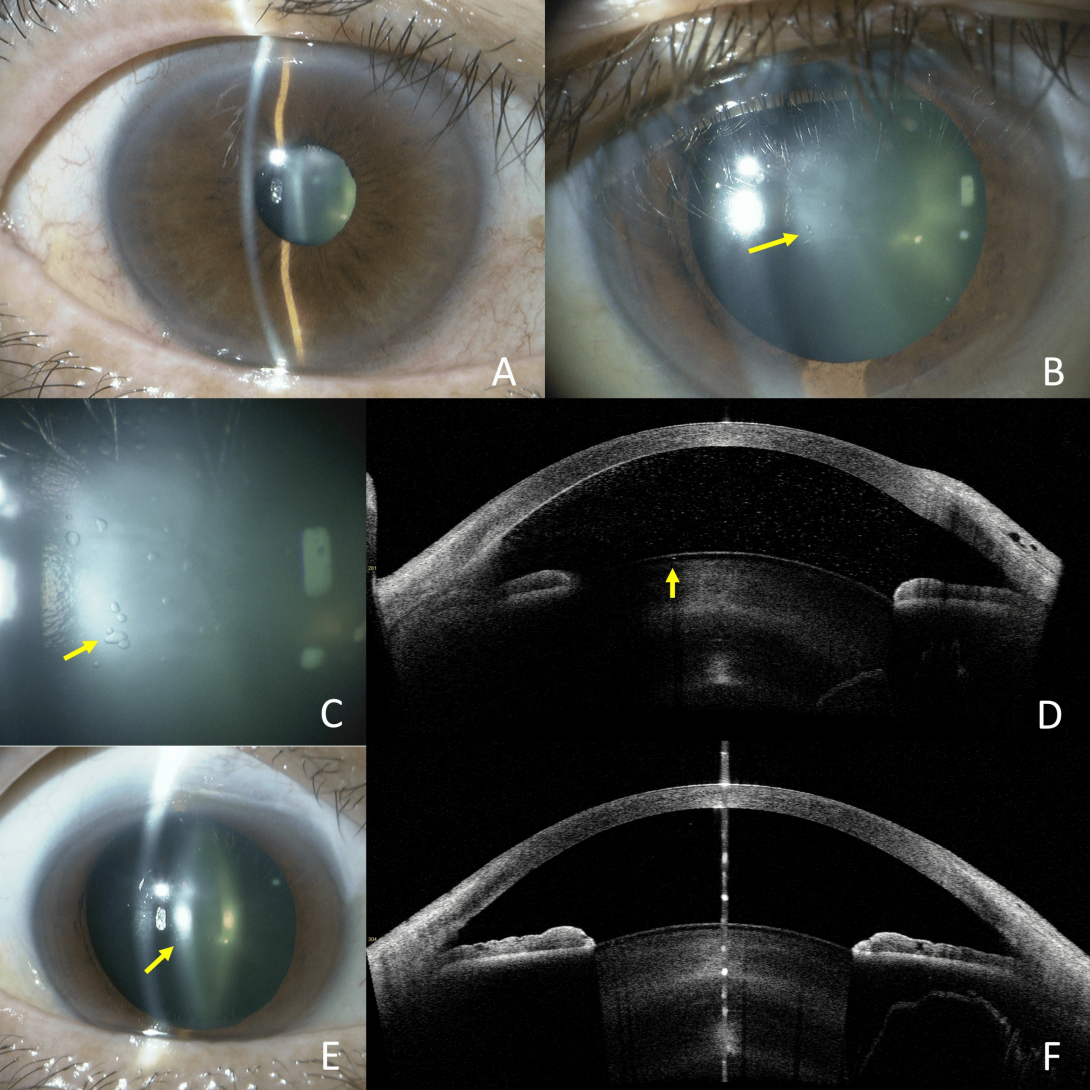
Case 5. Slit-lamp photography and anterior segment OCT imaging. Absence of anterior subcapsular cataract preoperatively (A). POD 7 showed vacuolar anterior subcapsular opacities of the lens (Arrow) (B). Opacities on a magnified image of the lesion (Arrow) (C). Only a few vesicles were detected on AS-OCT (Arrow) (D). Four months after surgery, the opacities almost disappeared (Arrow) (E) and had not been detected by AS-OCT (F). POD: Post-operative day; AS-OCT: Anterior segment optical coherence tomography

A 66-year-old man underwent trabeculectomy for POAG in the right eye. Preoperatively, there were no anterior subcapsular cataracts (Fig 5A), and the decimal BCVA was 1.0. The patient had a myopia of -5.0 diopters. On POD 7, vacuolar lens opacities were slightly observed (Fig 5B, 5C), and the decimal BCVA had decreased to 0.2. Only a few vesicles were detected on AS-OCT (Fig 5D). By eight months postoperatively (final visit), the vacuolar lens opacities had almost completely resolved (Fig 5E), and the decimal BCVA had improved to 0.8. Vesicles were not detected by AS-OCT (Fig 5F).

Table 1 summarizes the details of the cases.

**Table 1:** Summary of cases. M: Male; F: Female; JOAG: Juvenile open-angle glaucoma; SOAG: Secondary open-angle glaucoma; POAG: Primary open-angle glaucoma; OVD: Ophthalmic viscosurgical device;  BCVA: Best-corrected visual acuity

Case No.	Age (years)	Sex	Diagnosis	Preoperative refraction (Diopter)	OVD	Subcapsular opacity at the final visit	Preoperative BCVA (decimal)	BCVA at POD7 (decimal)	BCVA at final visit (decimal)	Follow-up period after operation (months)
1	42	M	JOAG	-10.00	Shellgan	decrease	0.9	0.2	0.4	6
2	64	M	SOAG	-3.75	Shellgan	decrease	0.9	0.4	1.2	4
3	65	M	POAG	-9.25	Shellgan	decrease	1.2	0.6	1.2	8
4	60	M	SOAG	-4.75	Shellgan	decrease	1.2	0.2	1.2	4
5	66	M	POAG	-5.00	Shellgan	disappearance	1.0	0.2	0.8	8

## Discussion

We encountered five cases of characteristic subcapsular vacuolar opacities following trabeculectomy using dispersive OVDs. All cases involved myopic eyes, and the lens opacities developed in the early postoperative period, showing a tendency to decrease over time. On POD 7, most cases exhibited reduced visual acuity, which may have been influenced by significant IOP fluctuations in the early postoperative phase. However, the extent to which the opacities contributed to the decline in visual acuity remains unclear. At the final follow-up, cases 2, 3, and 4 maintained their preoperative visual acuity, whereas Cases 1 and 5 experienced a decline. The vacuolar lens opacities demonstrated a decreasing trend over time, suggesting the potential for further improvement with extended follow-up periods.

In recent years, we reported similar characteristics, transient postoperative opacities following microhook trabeculectomy. In that report, we hypothesized that the opacities were caused either by traumatic mechanisms due to intraoperative irrigation or using dispersive OVDs [8]. Similarly, in other intraocular procedures preserving the crystalline lens, such as implantable collamer lens (ICL) implantation surgery, similar postoperative cataracts have been reported, with intraoperative fluid dynamics suggested as a potential mechanism [9,10]. However, in the present cases, cataracts occurred despite the absence of irrigation during the surgery, strongly suggesting that the dispersive OVD itself is the causative factor. Supporting this, Zhou et al. reported that in persistent pupillary membrane removal surgery cases, characteristic anterior subcapsular opacities were observed when dispersive OVDs were used but not with cohesive OVDs [11].

Furthermore, in conventional cataract surgery, they injected dispersive OVDs in one eye and cohesive OVDs in the other, then gently irrigated both with a blunt cannula. Intraoperative OCT showed droplet-like opacities only in the eye treated with dispersive OVDs. Electron microscopy of the extracted anterior capsule confirmed intercellular spaces in the tight junctions only in cases with dispersive OVDs [11]. These findings indicate that dispersive OVDs can cause subcapsular opacities even when carefully irrigated. While the previous report accounted for the potential effects of intraoperative fluid dynamics, the current cases did not involve irrigation after OVD injection, further strengthening the argument that the dispersive OVD itself is responsible for the observed changes. In similar reports of lens opacities following ICL implantation, dispersive OVDs were also used, and fluid dynamics were suspected as the cause [9,10]. The precise mechanism by which dispersive OVDs induce subcapsular opacities in the anterior lens capsule remains unclear and warrants further investigation.

Potential alternative explanations, including direct instrument contact, drug toxicity, and perioperative factors, were evaluated but ultimately ruled out.

A limitation of this study is that similar surgical procedures did not result in anterior subcapsular opacities in all cases. Since these findings often resolve spontaneously over time, it is possible that some cases may have gone unnoticed, leaving the incidence rate unclear. Therefore, multivariate analysis regarding causative factors was not performed. All five cases were myopic, suggesting that myopia might have partially contributed to the onset. Although trabeculectomy with lens preservation is widely performed in myopic cases, there have been no reports of similar characteristic cataract development. Despite this limitation, this is the first report demonstrating that transient anterior subcapsular vacuolar opacities can occur following intraocular surgery using dispersive OVDs without irrigation.

## Conclusions

This report highlights a postoperative phenomenon observed in five cases of transient subcapsular vacuolar cataracts following trabeculectomy with dispersive OVDs. These findings emphasize the need to recognize potential complications associated with OVDs and their impact on ocular tissues. By presenting these cases, we aim to raise awareness of this underreported complication, providing clinicians with a basis for early detection and management. To reduce such risks, we recommend using cohesive OVDs instead of dispersive ones in lens-sparing surgeries, including trabeculectomy, ICL implantation, and ab interno minimally invasive glaucoma surgery.
